# Genomic Insights into the Colistin Resistant *mcr-*Carrying *Escherichia coli* Strains in a Tertiary Hospital in China

**DOI:** 10.3390/antibiotics11111522

**Published:** 2022-11-01

**Authors:** Guoli Li, Xinyang Li, Yuye Wu, Juan Xu, Fang He

**Affiliations:** 1 Department of Clinical Laboratory, Sir Run Run Shaw Hospital, Zhejiang University School of Medicine, Hangzhou 310016, China; 2 School of Public Health, Hangzhou Medical College, Hangzhou 310013, China; 3 Laboratory Medicine Center, Department of Clinical Laboratory, Zhejiang Provincial People’s Hospital, Affiliated People’s Hospital, Hangzhou Medical College, Hangzhou 310014, China

**Keywords:** *Escherichia coli*, *mcr*, whole-genome sequencing, plasmid-mediated, colistin

## Abstract

Colistin is an important antimicrobial agent in the treatment of infections caused by multidrug resistant (MDR) Gram-negative bacteria. The horizontal transfer of mobile colistin resistance gene (*mcr*) poses a major threat to the public health worldwide. In this study, a total of thirteen *mcr*-carrying *Escherichia coli* (MCREC) strains were recovered from a tertiary hospital in Zhejiang, China, between 2016 and 2019. The minimum inhibitory concentration (MIC) of antimicrobial agents, epidemiological characteristics, and transmission dynamics of *mcr*-carrying isolates were analyzed using antimicrobial susceptibility testing, whole-genome sequencing, S1 nuclease pulsed-field gel electrophoresis (S1-PFGE), and southern blotting analysis. All strains were discovered to be resistant to colistin, and the majority displayed MDR phenotype. However, none of the 13 MCREC strains were resistant to carbapenems. The 13 MCREC isolates were divided into 10 different STs, including ST744, ST156, ST453, ST410, ST57, ST131, ST7034, ST2599, ST457, and ST13239, in which ST13239 was discovered for the first time. Based on core genome single nucleotide polymorphism (cgSNP) analysis, no clear epidemiological link was discovered in these strains with the exception of EC2118 and EC3807, which differ by just one SNP. A total of 35 antimicrobial resistance genes which can be divided into 14 classes were identified from the 13 MCREC isolates. According to S1-PFGE and southern blotting analyses, all 13 MCREC strains had plasmid-mediated *mcr-1*, and nine of them carried conjugative plasmids. In conclusion, our study revealed the emergence and dissemination of colistin-resistant *E. coli* isolates carrying *mcr-1* in a Chinese hospital, which poses a potential risk to anti-infective therapy. More efforts should be taken to monitor the prevalence of *mcr-1*-carrying bacteria in China.

## 1. Introduction

*Escherichia coli* is a widespread pathogen that can cause a variety of infections in both humans and animals. The spread of antibiotic resistance genes (ARGs) by transposons and transmissible plasmids in clinical strains makes it more challenging to treat [1]. Polymyxins are a class of cationic polypeptide antibiotics produced by *Bacillus polymyxa.* There are five chemically distinct polymyxins—A, B, C, D, and E—of which only polymyxin E (colistin) and polymyxin B are used in clinical settings [2]. Colistin is a narrow-spectrum antibiotic that is effective against the majority of Enterobacterales and is used as a last resort antibiotic for MDR Gram-negative bacteria. When the positively charged colistin molecule binds to the negatively charged lipid A on the cell wall of Gram-negative bacteria, it causes the outflow of the cell’s contents and cell death [3]. Several retrospective studies have revealed colistin resistance in both humans and animals globally as a result of the overuse and misuse of colistin. Additionally, the emergence of colistin-resistant pathogens worldwide does not coincide with any prior exposure to the antibiotics, which raises the possibility of the horizontal transfer of colistin resistance [4]. The emergence of colistin resistance and its adaptability, existence, and prevalence poses a significant global pandemic risk for public health [5].

The primary mechanism for colistin resistance, which is well known, is horizontal gene transfer of the plasmid-mediated mobile colistin resistance gene (*mcr*). The *mcr-1* gene was the first one to be described in an *E. coli* strain in China [6]. The *mcr* genes are frequently carried by a variety of plasmids, including IncI2, IncX4, IncP, IncX, and IncFII types [5,7]. Furthermore, a number of mobile genetic elements in plasmids, such as insertion sequences (particularly IS*Apl1*), class 1–3 integrons, and transposons like Tn*6330*, Tn*As2*, and Tn*3*-family, were thought to be the primary drivers of horizontal gene transfer of the *mcr* genes [8,9].

Ten *mcr* homologues (*mcr*-1–10) have so far been identified, and *mcr-1* continues to be the main character and is widely distributed [10,11,12]. The MIC of colistin in *E. coli* have been shown to increase by 4 to 8-fold when the *mcr-1* gene is present, indicating that *mcr-1* alone can provide adequate resistance against colistin in *E. coli* and other Enterobacteriaceae [2,13,14]. It is important to monitor the spread of *mcr*-carrying *E. coli* (MCREC) in clinical settings. To actively combat the threat posed by MDR Gram-negative Enterobacteriaceae, we must monitor MCREC strains and research their prevalence in order to stop further spread of these pathogens.

In this study, 13 MCREC isolates from a tertiary hospital in China were collected from 2016 to 2019. Whole-genome sequencing (WGS), S1 nuclease pulsed-field gel electrophoresis (S1-PFGE), and southern blotting analysis were performed to explore the antimicrobial resistant genes, epidemiological characteristics, and transmission dynamics of *mcr*-carrying isolates.

## 2. Results

### 2.1. Clinical Data of MCREC Isolates

A total of 882 clinical *E. coli* isolates were collected between 2016 and 2019. Urine is the most common (57.9%) of the clinical specimens, which also include blood (22.1%), sputum (4.9%), pus (3.6%), bile (3.4%), ascitic fluid (1.8%), and others. We collected 13 MCREC isolates from 12 patients. These MCREC clinical isolates were cultured from sputum (2/13), urine (8/13), pus (2/13), and ascitic fluid (1/13). The percentage of MCREC was 2.9% in 2016 (2/70), 3.4% in 2017 (6/175), 0.9% in 2018 (3/339), and 0.7% in 2019 (2/298). From 2018, there was a discernible decline in the occurrence of MCREC. Clinical data of the 12 patients with MCREC infection are presented in Table 1. Six of the twelve patients were male and six were female. Six patients were in the 65–90 age range, and five were in the 50–65 age range. The 11 patients were all inpatients, with the exception of one child patient who was only six months old and received care in an outpatient setting. Two patients passed away during the research period, and the remaining 10 patients gradually recovered and were discharged.

### 2.2. Antimicrobial Susceptibility Profiles

**Figure 1:**
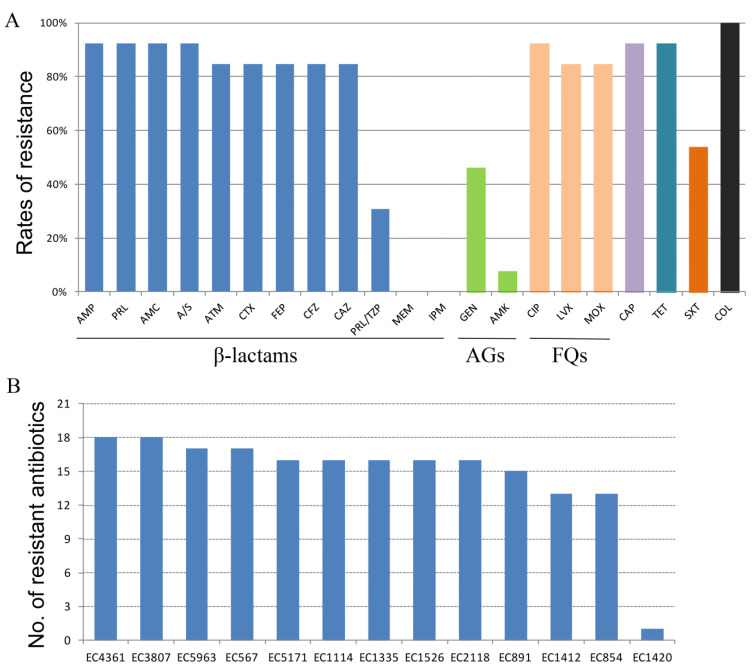
Antimicrobial susceptibility profiles of the 13 MCREC strains. (**A**) Resistance rates to 21 antimicrobial agents among the 13 MCREC strains. Each of the color represent one class of antibiotics; (**B**) The number of antibiotics that have been rendered ineffective for each MCREC strain. AGs, Aminoglycosides; FQs, Fluoroquinolones; AMP, ampicillin; PRL, piperacillin; ATM, aztreonam; CTX, cefotaxime; FEP, cefepime; MEM, meropenem; GEN, gentamicin; SXT, trimethoprim-sulfamethoxazole; LVX, levofloxacin; MOX, moxifloxacin; AMC, amoxicillin-clavulanic acid; A/S, ampicillin/sulbactam; PRL/TZP, piperacillin-tazobactam; CFZ, Cefazolin; CAZ, ceftazidime; IPM, imipenem; AMK, amikacin; CAP, chloramphenicol; TET, tetracycline; CIP, ciprofloxacin; COL, colistin. MCREC, *mcr*-carrying *Escherichia coli*.

A total of 21 antimicrobial agents—ampicillin (AMP), piperacillin (PRL), aztreonam (ATM), cefotaxime (CTX), cefepime (FEP), meropenem (MEM), gentamicin (GEN), trimethoprim-sulfamethoxazole (SXT), levofloxacin (LVX), moxifloxacin(MOX), amoxicillin-clavulanic acid (AMC), ampicillin/sulbactam (A/S), piperacillin-tazobactam (PRL/TZP), cefazolin (CFZ), ceftazidime (CAZ), imipenem (IPM), amikacin (AMK), chloramphenicol (CAP), tetracycline (TET), ciprofloxacin (CIP), and colistin (COL)—were used. Antimicrobial susceptibility profiles of the 13 MCREC strains are presented in Table 2. Figure 1A,B illustrate the rates of antibiotic resistance to the 21 antibiotics utilized in this investigation among the 13 MCREC strains, as well as the number of drugs that have been rendered ineffective for each MCREC strain. Of the 13 MCREC strains, 12 of them, except for EC1420, were MDR, which showed resistant to five to seven classes of antibiotics. Colistin resistance was present in all 13 MCREC strains, with MICs ranging from 4 to >64 mg/L. All 13 MCREC strains were susceptible to MEM and IPM, and the majority of them were also susceptible to AMK (92.3%, 12/13, except for EC854). EC4361 and EC3807 revealed the similar antimicrobial resistance profiles among the 21 antibiotics employed in this study, with the highest resistance to a total of 18 medications (except for MEM, IPM, and AMK). EC1526 and EC1420 were isolated from the same patient. EC1526 was found to be super resistant to colistin, with a MIC > 64 mg/L, and resistant to 16 antibiotics, whereas EC1420 was found to be only resistant to colistin but susceptible to the other 20 antibiotics.

### 2.3. Genomic and Phylogenetic Analysis of mcr-Carrying Isolates

**Figure 2:**
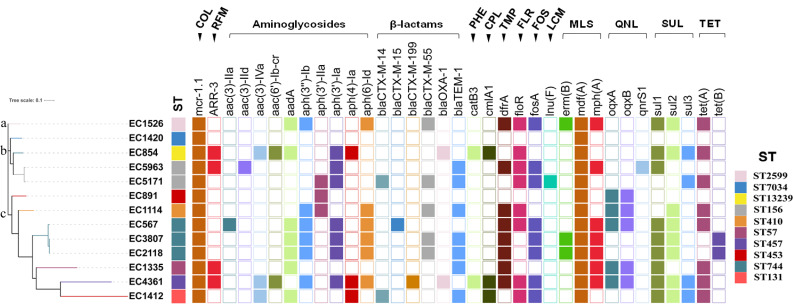
Phylogenetic relationship of the 13 MCREC isolates. The cell in different colour indicates the presence of the antimicrobial resistance gene while the blank cell indicates the absence of the gene. COL, colistin, RFM, rifamycin, PHE, phenicol, CPL, chloramphenicol, TMP, trimethoprim, FLR, florfenicol, FOS, fosfomycin, LCM, lincosamide, MLS, macrolides, QNL, quinolones, SUL, sulfonamides, TET, tetracyclines.

Antimicrobial resistance genes were identified and shown in Figure 2 based on WGS data from 13 MCREC isolates. A total of 35 antimicrobial resistance genes were identified, which can be classified into 14 classes, including one colistin-resistance gene (COL, *mcr-1*), six β-lactams-resistance genes (BLs, *bla*_CTX-M-14_, *bla*_CTX-M-15_, *bla*_CTX-M-199_, *bla*_CTX-M-55_, *bla*_OXA-1_ and *bla*_TEM-1_), ten aminoglycosides-resistance genes (AGs, *aac(3)-IIa*, *aac(3)-IId*, *aac(3)-Iva*, *aac(6’)-Ib-cr*, *aadA*, *aph(3’’)-Ib*, *aph(3’)-IIa*, *aph(3’)-Ia*, *aph(4)-Ia*, and *aph(6)-Id*), three sulfonamides-resistance genes (SUL, *sul1*, *sul2* and *sul3*), two tetracyclines-resistance genes (TET, *tet(A)* and *tet(B)*), one trimethoprim-resistance gene (TMP, *dfrA*), one fosfomycin-resistance gene (FOS, *fosA*), three macrolides-resistance genes (MLS, *erm(B)*, *mdf(A)* and *mph(A)*), three quinolones-resistance genes (QNL, *oqxA*, *oqxB* and *qnrS1*), one rifamycin-resistance gene (RFM, *ARR-3*), one florfenicol-resistance gene (FLR, *floR*), one chloramphenicol-resistance gene (CPL, *cmlA1*), one phenicol-resistance gene (PHE, *catB3*), and one lincosamide-resistance gene (LCM, *lnu(F)*). Colistin-resistance gene *mcr-1* was found in all 13 MCREC isolates. Except for EC1420, all of the other 12 MCREC isolates had at least one BL and one AG antimicrobial resistance gene. EC1420 was found to have only two resistance genes, *mcr-1* and *mdf(A)*, which is consistent with its antibiotic susceptibility profile (susceptible to most antibiotics). SUL were found in 11 MCREC isolates, while TET were found in 10 MCREC isolates. On the other hand, only EC5171 possessed LCM *(lnu(F)*), EC4361 and EC854 possessed PHE (*catB3*), and EC4361, EC854, and EC1412 possessed CPL (*cmlA1*). The presence of up to 24 genes encoding resistance to 13 classes of antibiotics (except LCM) in EC4361 is consistent with its antimicrobial resistance profile (resistant to most antibiotics).

Based on the phylogenetic tree (Figure 2), the 13 MCREC isolates were divided into three clades. Only EC1526 is in clade A, EC1420, EC854, EC5963, and EC5171 are in clade B, and the remaining eight MCREC isolates are in clade C. SNP analyses revealed that all of the MCREC isolates were sporadic, except for EC2118 and EC3807, which differ by only one SNP. MLST analyses of the 13 MCREC isolates revealed significant diversity, with the isolates assigned to 10 distinct STs, including three strains for ST744, two strains for ST156, and one strain for ST453, ST410, ST57, ST131, ST7034, ST2599, ST457, and ST13239, among which ST13239 is identified for the first time.

### 2.4. Conjugation Assay and Plasmid Analysis

**Figure 3:**
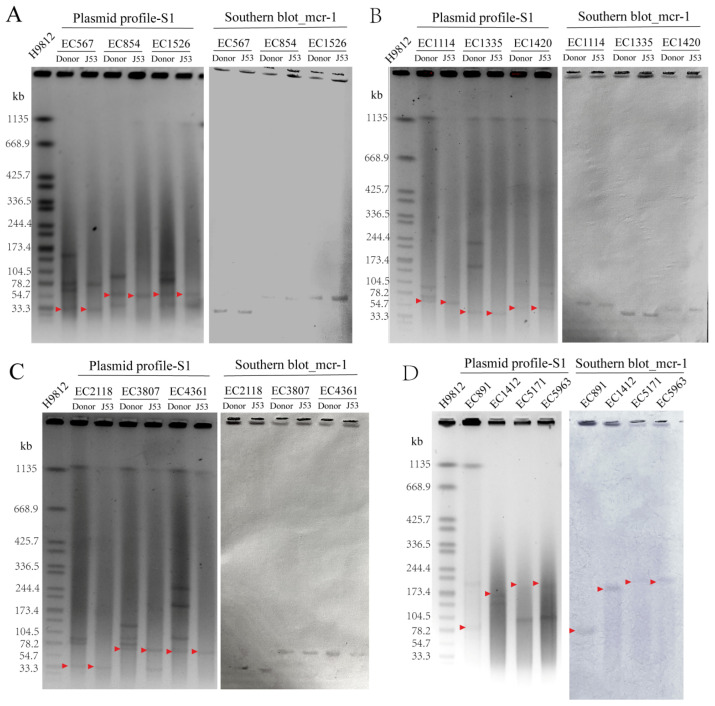
S1-PFGE and southern blotting for investigating plasmids containing *mcr*-1. MCREC isolates carrying conjugative *mcr*-1 plasmids were examined in panels (**A**–**C**). (**D**) Analysis of the four MCREC isolates that carrying nonconjugative *mcr-1* plasmids. The *Salmonella* strain (H9812) was used as a marker. Red arrows represent the *mcr-1*-carrying plasmids.

The transferability of *mcr-1*-carrying plasmids from the 13 MCREC isolates were determined by conjugation experiment. S1-PFGE and southern blotting analysis were performed to estimate the size of the *mcr-1*-carrying plasmid. Nine out of the thirteen MCRECs, with the exception of EC5963, EC5171, EC891, and EC1412, have conjugative plasmids carrying the *mcr-1* gene. The conjugation efficiency is calculated with the high conjugation efficiency of 8.6 × 10^−2^ for EC1335, 7.3 × 10^−2^ for EC2118, and 1.1 × 10^−2^ for EC3807, the middle conjugation efficiency of 2.1 × 10^−3^ for EC1526, 2.1 × 10^−3^ for EC854, 1.8 × 10^−3^ for EC1114, 8.0 × 10^−4^ for EC4361, and the low conjugation efficiency of 6.3 × 10^−5^ for EC1420, 3.5 × 10^−6^ for EC567. Figure 3 depicts the S1-PFGE and southern blot results. The size of *mcr-1*-carrying plasmids was heterogeneous, ranging from 30 kb to 250 kb. The size of *mcr-1*-carrying plasmids in the nine conjugative MCREC isolates can be divided into two types: the smaller one is between 33.3kb and 54.7 kb (for EC1420, EC567, EC2118, and EC1335), while the larger one is between 54.7 kb and 78.2 kb (for EC1526, EC854, EC1114, EC3807, EC4361). All of the *mcr-1-*carrying plasmids in the four nonconjugative MCREC isolates were between 80 kb and 250 kb in size. Furthermore, EC567, EC1526, and EC1420 harbored two types of plasmids of varying sizes that can be conjugative. According to WGS, the 13 MCREC plasmids were divided into three plasmid incompatibility groups: seven IncI2 (EC854, EC1114, EC1420, EC1526, EC4361, EC5171, EC5963), five IncX4 (EC567, EC891, EC1335, EC2118, EC3807), and one IncHI2 (EC1412).

## 3. Materials and Methods

### 3.1. Patients and Isolates

Between January 2016 and October 2019, a total of 882 *E. coli* clinical isolates were collected from patients at a tertiary hospital in Zhejiang province, China. Using the previously published primers, a standard PCR was used to detect the *mcr-1* gene [6]. A total of 13 *mcr-1* positive *E. coli* isolates from 12 patients were identified. Ascitic fluid, pus, urine, and sputum were among the specimens used to cultivate the isolates. All isolates were generated as part of routine clinical laboratory procedures. The patient administration system was used to extract demographic information, such as gender, age, the hospital department where the patient was admitted, the clinical diagnosis, and the outcome, without obtaining any personally identifiable information about the patient. This study was previously approved by the Research Ethics Committee of Zhejiang Provincial People’s Hospital.

### 3.2. Species Identification and Antimicrobial Susceptibility Testing

All isolates were identified using the MALDI-TOF-MS system (Bruker, Billerica, MA, USA). Antimicrobial susceptibility testing was conducted using standard broth microdilution tests following the guidelines of the Clinical and Laboratory Standards Institute (CLSI). *E. coli* ATCC 25922 was used as a quality control. The European Committee on Antibiotic Susceptibility Testing (EUCAST, https://www.eucast.org/ accessed on 16 July 2021) 12.0 guidelines were used to interpret the susceptibility breakpoint for colistin and isolates with MIC > 2 mg/L were interpreted as resistance. CLSI 2021 guidelines were used to interpret the susceptibility breakpoint for other antimicrobial agents [15]. MDR isolates were identified as those that were simultaneously resistant to three or more classes of antibiotics.

### 3.3. Whole-Genome Sequencing (WGS)

Isolates confirmed to carrying the *mcr* gene were sent for whole-genome sequencing using the Illumina NovaSeq 6000 platform (Illumina Inc., San Diego, CA, USA). In brief, genomic DNA was isolated using a QIAamp DNA MiniKit (Qiagen, Valencia, CA, USA) and sent for sequencing according to the paired-end 2 × 150-bp protocol. SPAdes 3.13.0 was used to assemble the draft genome sequences, and the NCBI Prokaryotic Genome Annotation Pipeline (PGAP) server was used to automatically annotate the genome sequences [16].

### 3.4. Genomic and Phylogenetic Relationship Analysis of mcr-Carrying Isolates

The MLST, acquired ARGs, and plasmid replicons of the *mcr*-carrying isolates were analyzed using the BacWGSTdb 2.0 server [17,18,19]. The phylogenetic relationship between *mcr-*carrying isolates was analyzed using the neighbor joining (NJ)/unweighted pair group method with arithmetic mean (UPGMA) phylogeny method (MAFFT version 7) based on a core genome single nucleotide polymorphism strategy [20]. The resulting SNPs were used to build a phylogenetic tree, and the maximum parsimony algorithm was used to remove recombination regions [21]. The Interactive Tree Of Life (iTOL) v6 web server was utilized for the phylogenetic tree visualization and annotation of ARGs [22].

### 3.5. Conjugation Experiments and Southern Blot

The transferability of *mcr*-carrying plasmids from isolates was assessed using filter mating with *E. coli* J53 as the recipient strain, and the transconjugants were chosen on BHI agar plates supplemented with 2 mg/L colistin. The *mcr-*1 gene was then detected in transconjugants by screening and sequencing, as previously described [23]. The conjugation efficiency was measured and calculated following the protocol in https://openwetware.org/wiki/conjugation, accessed on 16 July 2021. A *Salmonella* serotype Braenderup strain (H9812) was chosen as the common size standard [24]. S1-nuclease PFGE (S1-PFGE) was used to measure plasmid sizes, and southern blot analysis with *mcr* gene-specific probes was used to pinpoint the genetic location of *mcr*-1, as previously described [25].

### 3.6. Nucleotide Sequence Accession Numbers

We deposited the genome sequences of the *mcr-1* positive *E. coli* strains in NCBI GenBank under the BioProject accession number PRJNA515159 (JANYUL000000000-JANYUX000000000).

## 4. Discussion

In China, *E. coli* is the most common species carrying plasmid-mediated colistin resistance in the Enterobacteriaceae [6]. In this study, we used PCR to screen for the presence of the gene *mcr-1* in 882 *E. coli* strains that were isolated from human clinical samples in Zhejiang Province, China, between 2016 and 2019. These samples included sputum, urine, pus, and ascitic fluid. MCR-1 positivity and colistin resistance were found in 13 of the *E. coli* strains. Notably, the *mcr-1* carriage rate dropped from 2.9% and 3.4% in 2016 and 2017 to 0.9% and 0.7% in 2018 and 2019. Colistin use in animal farming is thought to be a major driver in the dissemination of colistin resistance [6]. The decline in *mcr-1* carriage rate may be related to the policy that the Ministry of Agriculture of China (Article number 2428) withdrew colistin as a feed additive and growth promoter in November 2016 and formally implemented in April 2017 [26]. Several other studies also indicated that colistin resistance reached peak around later 2016–mid 2017 in China because of the regulated use of colistin, which indicated that the banning had a significant effect on reducing colistin resistance in both animals and humans [27,28,29,30]. As colistin was thought to be a last resort for the treatment of serious Gram-negative bacterial infections, therefore, despite the fact that the spread of colistin resistance is slowing as a result of the ban, monitoring and tracking colistin resistance in Enterobacteriaceae is crucial, and additional investigation is still required to fully assess the molecular epidemiology of plasmid-mediated colistin resistance in Enterobacteriaceae.

Due to its high capacity to accumulate ARGs, *E. coli* has been reported to be a significant reservoir of ARGs [31]. Additionally, it has been established that *E. coli*, primarily through horizontal gene transfer, plays critical roles in the spread of the *mcr* genes [6]. In this study, the majority of MCREC strains also exhibited MDR with a high rate of resistance to common antibiotics used in clinical settings, such as ampicillin, piperacillin, aztreonam, cefotaxime, cefepime, levofloxacin, moxifloxacin, amoxicillin-clavulanic acid, ampicillin/sulbactam, cefazolin, ceftazidime, chloramphenicol, tetracycline, and ciprofloxacin. All 13 MCREC strains were colistin resistant and carried the *mcr-1* gene on plasmids. However, MICs of these MCREC strains varied greatly, ranging from 4 to >64 mg/L. This has led us to speculate on the existence of other mechanisms, such as different expression activities of different *mcr*-carrying plasmid types or different genomic background of the MCREC strains which might possess active efflux pump or particular genetic mutations, and so on. Deeper sequencing and further analysis will give more information about the different mechanisms of colistin resistance.

In particular, the isolates EC1420 and EC1526 were taken from the same patient Pa1420, who passed away during this study, but the two strains were dispersed sporadic ones. The later isolate EC1526 showed super resistance to colistin with the MIC exceeding 64 mg/L and showed resistance to 16 antibiotics, whereas the earlier isolate EC1420 carried the fewest resistance genes and only demonstrated colistin resistance with a MIC of 8 mg/L. In addition, the plasmid carrying *mcr-1* in EC1420 was the smaller one, ranging from 33.3 to 54.7 kb, and it demonstrated the lowest transferability frequency, whereas the plasmid carrying *mcr-1* in EC1526 was the larger one, ranging from 54.7 to 78.2 kb, and it demonstrated a higher transferability frequency. It is possible that EC1526, not EC1420, was responsible for Pa1420’s final death from severe septicopyemia. However, further investigation is still required to identify the mechanisms underlying the extreme colistin resistance of EC1526. It was fortunate that none of the MCREC isolates were carbapenem-resistant isolates and that none of the 13 MCREC isolates contained any carbapenem-resistant genes.

WGS identified three groups of plasmids from the 13 MCREC, including IncI2, IncX4, and IncHI2. Despite the fact that we did not examine the complete plasmid sequences, we used S1-PFGE and southern blotting analysis to determine the size of the plasmids and the transferability of plasmids containing the *mcr-1* gene. The plasmids were found to be between 30 and 250 kb in size. The plasmid carrying the transferable *mcr-1* gene was present in nine of the thirteen MCREC isolates. It should be noted that the genome sequences of EC2118 and EC3807 differ only by one SNP, despite coming from different patients and being collected over nine months apart. Their plasmid profiles vary greatly from each other, which may be the cause of their various antimicrobial resistance profiles. It was hypothesized that EC2118 and EC3807 shared a common ancestor but had different plasmids when they underwent horizontal transfer.

## 5. Conclusions

In summary, our study revealed the emergence and dissemination of colistin-resistant *E. coli* isolates carrying *mcr-1* in China, provided some genomic characteristics of *mcr*-harboring plasmids, and emphasized their role in dissemination of antimicrobial resistance in clinical settings. More efforts should be taken to monitor the prevalence of mcr-1 in a One Health approach.

## Figures and Tables

**Table 1:** Clinical data and outcomes of the 12 inpatients with *mcr*-carrying *E. coli* infection.

Patient	Isolates	Age	Gender	Department	Diagnosis	Outcome	Isolation Date	Specimens
Pa567	EC567	70	Male	Hepatology	Liver cirrhosis; History of gastric carcinoma	Died	31 May 2016	Ascitic fluid
Pa854	EC854	63	Male	General surgery	Abdominal Abscess	Discharged	25 December 2016	Pus
Pa891	EC891	79	Female	Urinary surgery	Ureteral calculus	Discharged	9 January 2017	Urine
Pa1114	EC1114	0.5	Female	Pediatrics	Fever	Discharged	20 April 2017	Urine
Pa1335	EC1335	62	Female	General surgery	Adenomyomatosis of gallbladder	Discharged	26 June 2017	Pus
Pa1412	EC1412	70	Male	Endocrinology	Type 2 diabetes	Discharged	13 July 2017	Urine
Pa1420	EC1420	76	Female	Intensive care unit	Severe sepsis	Died	12 July 2017	Urine
	EC1526						11 August 2017	Urine
Pa2118	EC2118	54	Female	Nephropathy	Acute kidney injury	Discharged	13 January 2018	Urine
Pa3807	EC3807	52	Female	Urinary surgery	Urinary tract infection	Discharged	29 October 2018	Urine
Pa4361	EC4361	70	Male	Respiratory	Pulmonary infection; Pulmonary malignancy	Discharged	26 December 2018	Sputum
Pa5171	EC5171	88	Male	Intensive care unit	Pulmonary infection	Discharged	21 April 2019	Sputum
Pa5963	EC5963	50	Male	Neurosurgery	Multiple myeloma; Pulmonary infection	Discharged	16 August 2019	Urine

**Table 2:** Minimal inhibitory concentrations (MICs, mg/L) of the 13 *mcr*-carrying *E. coli* isolates to different antimicrobials.

Isolate	MIC (mg/L)																				
	β-Lactams												AGs		Fluoroquinolones			Others			
	AMP	PRL	ATM	CTX	FEP	MEM	IPM	AMC	A/S	PRL/TZP	CFZ	CAZ	AMK	GEN	LVX	MOX	CIP	SXT	CAP	TET	COL
EC567	>16	>64	>16	>32	16	≤1	≤1	8/4	16/8	≤4/4	>16	8	≤8	>8	>8	>4	>2	>2/38	>16	>8	8
EC854	>16	64	≤2	≤1	≤2	≤1	≤1	16/8	>16/8	16/4	≤4	≤1	16	>8	>8	>4	>2	≤0.5/9.5	>16	>8	8
EC891	>16	>64	>16	>32	>16	≤1	≤1	8/4	16/8	≤4/4	>16	16	≤8	≤2	>8	>4	>2	≤0.5/9.5	>16	>8	4
EC1114	>16	>64	>16	>32	>16	≤1	≤1	8/4	>16/8	≤4/4	>16	16	≤8	≤2	>8	>4	>2	>2/38	>16	>8	4
EC1335	>16	>64	>16	>32	>16	≤1	≤1	8/4	16/8	≤4/4	>16	>16	≤8	≤2	2	4	2	>2/38	16	>8	4
EC1412	>16	>64	8	>32	>16	≤1	≤1	16/8	>16/8	≤4/4	>16	2	≤8	>8	≤1	≤1	≤0.5	≤0.5/9.5	>16	>8	8
EC1420	≤4	≤4	≤2	≤1	≤2	≤1	≤1	≤4/2	≤4/2	≤4/4	≤4	≤1	≤8	≤2	≤1	≤1	≤0.5	≤0.5/9.5	≤4	≤2	8
EC1526	>16	>64	>16	>32	>16	≤1	≤1	16/8	>16/8	≤4/4	>16	>16	≤8	≤2	>8	>4	>2	>2/38	>16	>8	>64
EC2118	>16	>64	>16	>32	>16	≤1	≤1	8/4	8/4	≤4/4	>16	8	≤8	≤2	>8	>4	>2	>2/38	8	>8	4
EC3807	>16	>64	8	>32	>16	≤1	≤1	16/8	>16/8	>64/4	>16	16	≤8	>8	>8	>4	>2	1/19	8	>8	8
EC4361	>16	>64	8	>32	>16	≤1	≤1	16/8	>16/8	>64/4	>16	>16	≤8	>8	>8	>4	>2	>2/38	>16	>8	4
EC5171	>16	>64	>16	>32	>16	≤1	≤1	16/8	>16/8	>64/4	>16	>16	≤8	≤2	>8	>4	>2	≤0.5/9.5	>16	>8	4
EC5963	>16	>64	>16	>32	>16	≤1	≤1	16/8	>16/8	≤4/4	>16	>16	≤8	>8	>8	>4	>2	>2/38	>16	>8	4

Abbreviations: AGs, Aminoglycosides; AMP, ampicillin; PRL, piperacillin; ATM, aztreonam; CTX, cefotaxime; FEP, cefepime; MEM, meropenem; IPM, imipenem; AMC, amoxicillin-clavulanic acid; A/S, ampicillin/sulbactam; PRL/TZP, piperacillin-tazobactam; CFZ, Cefazolin; CAZ, ceftazidime; AMK, amikacin; GEN, gentamicin; LVX, levofloxacin; MOX, moxifloxacin; CIP, ciprofloxacin; SXT, trimethoprim-sulfamethoxazole; CAP, chloramphenicol; TET, tetracycline; COL, colistin.

## Data Availability

Data is contained within the article.
